# Hybrid Aortic Arch Replacement with Frozen Elephant Trunk (FET) Technique: Surgical Considerations, Pearls, and Pitfalls

**DOI:** 10.3390/jcm13237075

**Published:** 2024-11-22

**Authors:** Dimos Karangelis, Theodora M. Stougiannou, Konstantinos C. Christodoulou, Henri Bartolozzi, Maria Eleni Malafi, Fotios Mitropoulos, Dimitrios Mikroulis, Martin Bena

**Affiliations:** 1 Department of Cardiothoracic Surgery, Democritus University of Thrace University General Hospital, 68100 Alexandroupolis, Greece; theodorastougiannos@gmail.com (T.M.S.); henribartolozzi02@gmail.com (H.B.); marilenmalafi@gmail.com (M.E.M.); dmikrou@med.duth.gr (D.M.); 2 Center for Thrombosis and Hemostasis (CTH), University Medical Center of the Johannes Gutenberg University, 55131 Mainz, Germany; konstantinoschristodoulou@yahoo.gr; 3 Department of Adult and Congenital Cardiac Surgery, Mitera Hospital, 15123 Athens, Greece; fotiosmitropoulos@yahoo.com; 4 Department of Cardiac Surgery, CINRE Hospital, 84103 Bratislava, Slovakia; martinbena.mb@gmail.com

**Keywords:** aortic arch, thoracic aortic aneurysm, aortic dissection, frozen elephant trunk, prosthesis, stent, graft

## Abstract

The involvement of the aortic arch in thoracic aortic aneurysms (TAA), or acute aortic dissections (AAD), represents a challenging clinical entity, mandating a meticulous surgical plan, tailored to each individual case. The advent of endovascular techniques and the introduction of modern arch protheses have led to the implementation of the frozen elephant trunk (FET) technique. This one-step hybrid operation consists of a total aortic arch replacement combined with an antegrade delivery of a stent–graft for the descending aorta, which acts as a proximal landing zone facilitating a potential distal endovascular reintervention. In this manner, this technique addresses acute and chronic arch disease with an acceptable morbidity and mortality. Several FET prosthetic devices are available on the global market and have exhibited favourable outcomes, although with some disadvantages in complex cases; similarly, the hybrid procedure described in this review has also been associated with complications, such as coagulopathy and neurological and graft-related events. The purpose of this review is to thus provide key insights into successful hybrid aortic arch replacements and to discuss useful tips and relevant considerations regarding its use.

## 1. Introduction

A thoracic aortic aneurysm (TAA) is defined as a localized dilatation of the thoracic aorta (TA), a disease with a silent nature and that, in most instances, is asymptomatic. Although recent systematic reviews and meta-analyses have reported the incidence of TAA to be around 5–10 per 100,000 individuals per year, its true incidence remains uncertain, with the relevant epidemiological data understudied, even though some additional data have shown it to be increasing [1,2,3]. The lack of specific screening programs often render it misdiagnosed or completely unidentified. TAA, along with acute aortic dissection (AAD), are generally described as the two principal aortic diseases, sharing many common principles and repair techniques; their management, especially in emergency cases that involve the aortic arch, still remains a surgical challenge, often requiring a well-orchestrated multidisciplinary collaboration. In this regard, contemporary advancements in surgical techniques, including composite grafts with a frozen elephant trunk (FET), have brought about improvements and made aortic arch surgery possible, along with the concurrent management of the descending thoracic aorta. The FET allows for a one-step hybrid operation, and it is an innovative approach for complex aortic disease, as it integrates the traditional elephant trunk operation described by Borst [4] alongside an endovascular stent deployment in the descending aorta. The indications for an FET procedure have thus expanded to include aneurysms of the aortic arch and proximal descending thoracic aorta; acute and chronic type A aortic dissections with expansion in the aortic arch; acute and chronic type B dissections in cases where endovascular procedures are not indicated; and non-A, non-B acute aortic dissections [5]. Worldwide, FET procedures are continuously gaining in popularity, with recent projections from the Society of Thoracic Surgery (STS) expecting the number of implantations to grow significantly [6,7]. The commercially available devices which are the most commonly used include the E-vita^®^ Open Neo by Artivion (GA, USA) (formerly Cryolife/Jotec, JOTEC GmbH, Lotzenäcker 23, 72379 Hechingen, Germany) and the Thoraflex^TM^ by Terumo Aortic (Glasgow, Scotland). With regards to the Thoraflex^TM^ graft, it seems to be extensively utilized internationally, and while it has become the world’s first multibranched “frozen elephant trunk (FET)” prosthesis after receiving CE Mark approval in 2012, it was not until 2020 that it received FDA approval as well [8].

There are several tips, considerations, and repair pearls to take into account when utilizing such a graft for aortic arch replacement via the FET technique; it is the aim of this short narrative review to summarize some of these, particularly with regard to the Thoraflex^TM^ graft.

## 2. Cannulation Strategy and Cerebral Protection

**Figure 1:**
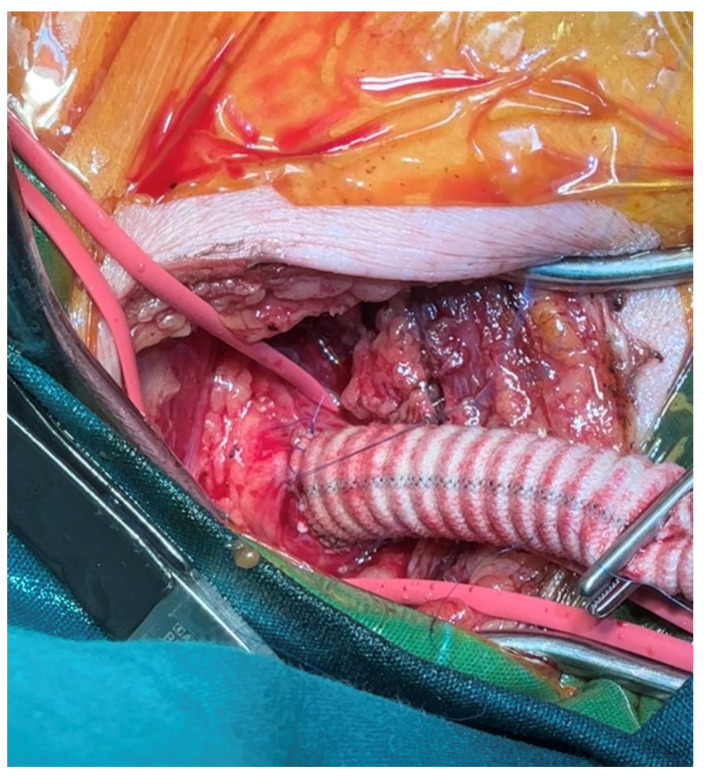
Intraoperative image of 8 mm graft anastomosed to right axillary artery.

The selection of the arterial cannulation site for a total arch replacement remains debatable, though several sites have been proposed with various advantages and disadvantages. Thus, the cannulation strategy must be specifically tailored to each case. The majority of the existing data derived from observational studies have supported the use of a right axillary artery cannulation; however, this suggestion has yet to be confirmed by randomized controlled trials [9]. We, as well, favour this technique as the standard approach in most cases, as it facilitates the use of an antegrade cerebral perfusion and has proved beneficial compared to a femoral artery cannulation, in terms of the mortality rates and neurological complications, especially for type A aortic dissections [10]. Additionally, this approach remains associated with retrograde flow as per anatomical considerations, with less travelling compared to a femoral cannulation. Studies have claimed that true antegrade flow can be achieved by the direct cannulation of the innominate and left common carotid artery or by the direct anastomosis of a 10 mm Dacron graft to each common carotid artery following two small incisions at the base of the neck [11,12]. In an effort to reduce limb ischemia rates, we typically deploy an 8 mm Dacron prosthesis anastomosed via an end-to-side fashion to the right axillary artery (Figure 1).

Though this technique has been criticized as time consuming, requiring a second incision, being anatomically more complex, and potentially associated with various complications, such as a brachial plexus injury, our experience has shown that in hemodynamically stable patients, it seems to be relatively safe and does not necessarily prolong an operation. In cases where the tissue quality of the innominate artery is acceptable, the cannulation of the innominate may also be considered. Another option that is often preferred due to its convenience and ease of establishment is the cannulation of the femoral artery. It may also be employed in cases with hemodynamic instability requiring urgent mechanical support, though it has been associated with higher rates of postoperative transient neurological dysfunction, as it cannot properly guarantee antegrade cerebral perfusion. Venous drainage is usually accomplished via a two-stage venous cannula in the right atrium, while a vent within the right superior pulmonary artery is used as well [13].

Cerebral protection during an aortic arch surgery is a well-known and active area of continuous research; it is of paramount importance during surgery, as the risk of neurological injury necessitates a meticulous strategy for neuroprotection. The pharmacologic neuroprotection and topical cooling applied by the anesthesiologist, as adjunctive measures to hypothermia, are equally important. Deep hypothermic circulatory arrest (DHCA) is crucial to minimize end-organ injuries, especially cerebral injuries, by decreasing the cerebral metabolic activity. As our group has previously shown, DHCA still has some drawbacks; thus, we advocate for moderate hypothermic circulatory arrest (26 °C to 28 °C), in conjunction with antegrade cerebral perfusion. In a recent meta-analysis, this combination was associated with significantly better postoperative outcomes, primarily in terms of the mortality and stroke rates, compared to DHCA [14]. DHCA, first introduced by Griepp and colleagues, represents the cornerstone of neuroprotection in modern aortic arch surgery [15] and, when accompanied by antegrade cerebral protection, it is usually associated with reduced rates of perioperative stroke [16,17]. The cannulation of the axillary artery, followed by the clamping of the innominate, left common carotid, and subclavian, seems to be enough for the sufficient oxygenation of both cerebral hemispheres, with no need for a bilateral antegrade cerebral perfusion, provided the flow is maintained at 10–15 mL/kg/min [10,18,19].

## 3. Design of the Thoraflex^TM^ Hybrid Graft

**Figure 2:**
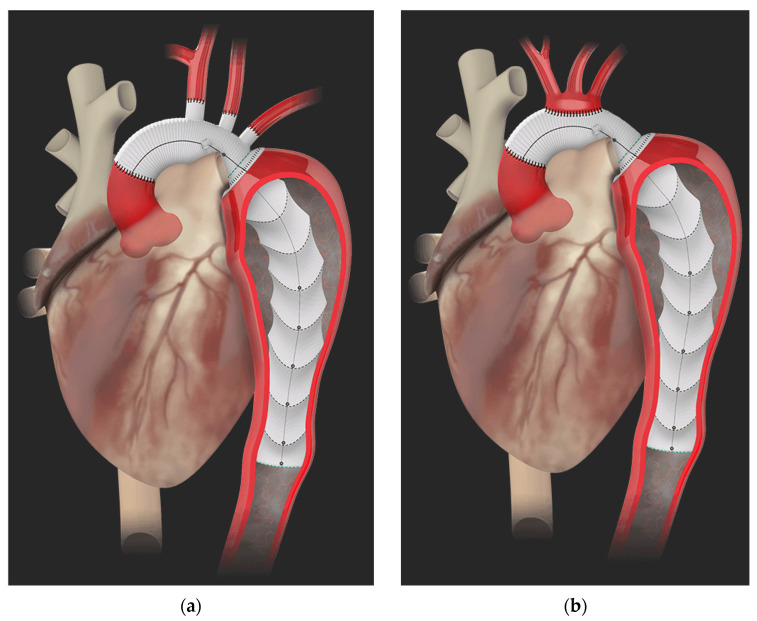
(**a**) Plexus^TM^ and (**b**) Ante-flo^TM^ designs. Figure obtained from Terumo Aortic [22].

The FET technique for a total arch replacement has revolutionized the planning and execution of this procedure; it offers a reliable platform, facilitates earlier patient rewarming, and reduces myocardial and lower-body ischemia, as well as operating times [20]. The Thoraflex^TM^ comes available with two possible designs, namely the Plexus^TM^ and the Ante-flo^TM^, for an individual arch vessel reconstruction or island technique, respectively. The Plexus^TM^ device comprises a multibranched arch prosthesis, including three branches for supra-aortic vessel reimplantation and one side branch for lower body perfusion (Figure 2a), while the Ante-flo^TM^ graft is designed to incorporate a single perfusion side arm (Figure 2b). Both grafts further incorporate a self-expanding stent, which is deployed in an open antegrade fashion, once the sheath is retracted back through the splitter [20,21]. A Gelweave^TM^ Siena collar is located distally to the perfusion branch at the location of the distal aortic anastomosis [21], allowing for easier and safer anastomosis of the prosthesis to the aorta, while reducing the haemodynamic traction on the anastomosis as well. The stent itself comes with radiopaque markers to simplify secondary procedures, if needed [20]. In general, the graft offers a unique design with independent, ring-shaped stents that promote better adaptation into the descending thoracic aorta, reducing the radial forces applied to the aortic wall, and thus mitigating the risk of intimal injury in AAD patients; it is available in different sizes (28–40 mm in diameter for the stented portion), with length of the stented segment either 100 or 150 mm [21]. The longer coverage of the descending aorta is hypothesized to affect the rate of spinal complications [6] and in this regard, we, along with other authors [6], advocate the use of the 100 mm stent graft length.

## 4. Surgical Technique

**Figure 3:**
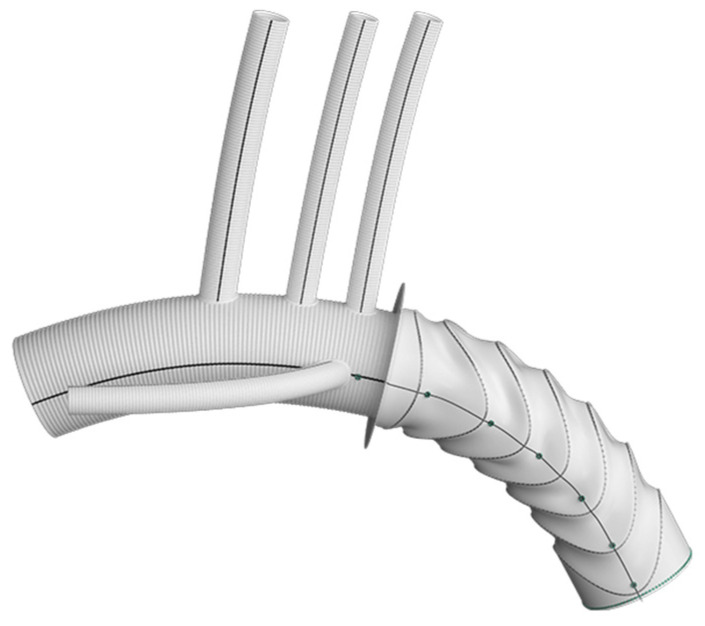
Thoraflex^TM^ Hybrid device 4-branch design. Figure obtained from Terumo Aortic [22].

Reiterating these next steps is essential and beneficial, especially for younger or inexperienced colleagues. After the establishment of cardiopulmonary bypass (CPB) and the placement of a left ventricle vent within the right superior pulmonary vein, cooling is initiated to achieve mild hypothermia (26 °C to 28 °C). During cooling, it is advisable to prepare the supra-aortic vessels. Once a cross clamp is applied, Custodiol^®^ cardioplegia is delivered into the aortic root. At this point, it is vitally important to mention that a detailed analysis of the aortic arch and supra-aortic vessels is essential for proper graft anastomosis, which we usually perform within zone 2 of the aorta, as well as the preferential deployment of a shorter-length stent within the stented portion of the hybrid graft, to reduce the rates of spinal cord injury; zone 3 should be avoided to prevent a laryngeal nerve injury. Following the resection of the arch and head–neck vessels, the distal graft anastomosis is carried out with two 4-0 pledgeted sutures, on positions 3 and 9, with the surgical pledgets positioned outwards from the aortic wall, while the Thoraflex^TM^ black line should be sitting on position 12 (Figure 3). The stented portion of the Thoraflex^TM^ graft should be slightly bent, so that it may adapt and conform to the curvature of the descending thoracic aorta; its deployment should occur under direct vision within the downstream aorta, after which the distal anastomosis should be secured with a second, circumferential, 4-0 prolene suture, ensuring a complete seal.

**Figure 4:**
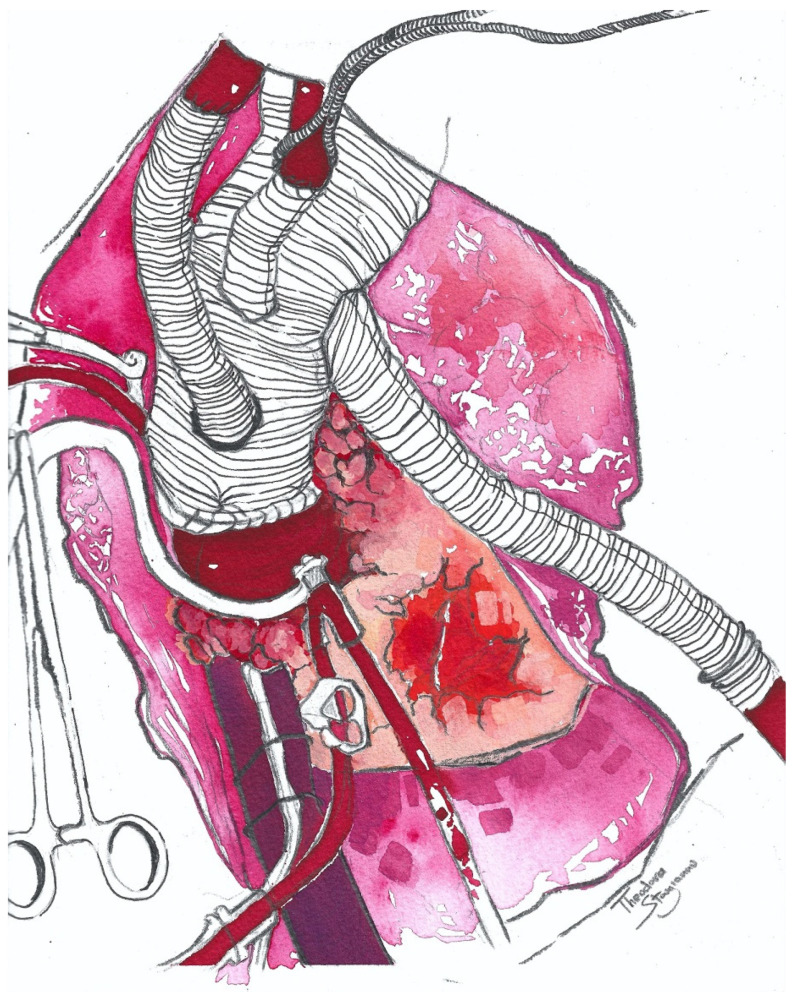
Illustration showing the Thoraflex^TM^ Hybrid graft with arch vessel reconstruction (left subclavian artery anastomosis is not depicted) and proximal anastomosis to the ascending aorta. The perfusion side graft is also shown.

Once the distal anastomosis is complete, the perfusion side branch is connected to the second limb of the aortic line and perfusion within the downstream aorta, through the elephant trunk, is initiated. Afterwards, the graft is de-aired and clamped, along with the side branches, and rewarming is commenced; at this point, cerebral perfusion is supplied through the 8 mm graft sutured to the axillary artery, while lower body perfusion is provided through the second arm of the aortic line. At this stage, the proximal anastomosis may be completed before the hand and neck vessels are anastomosed, so that myocardial perfusion can be reinstated, with the heart providing perfusion to the lower body, while at the same time, cerebral perfusion is still maintained by the axillary graft. Nevertheless, one may opt to perform the anastomoses of the supra-aortic vessels before the proximal anastomosis; in this case, the sequence in which the head vessels are reconstructed may vary depending on the native anatomy (Figure 4). The so-called “branch-first technique” has also been described, which entails the clockwise sequential ligation/disconnection and reconstruction of each arch branch (beginning with the innominate artery and ending with the left subclavian artery [LSA]), by employing a trifurcation graft with a side-arm port for perfusion [23,24].

A common consideration is the level of technical difficulty associated with reattaching the LSA; in elective cases, in which the preoperative imaging demonstrates a challenging local anatomy, including the LSA lying deep within the tissue plane, instead of a direct vessel-to-arch reattachment, the patient may be subjected to a left carotid artery (LCA) to subclavian artery (SC) bypass, via a ligation or coil embolization of the proximal LSA, or via a LCA-SC transposition [25].

**Figure 5:**
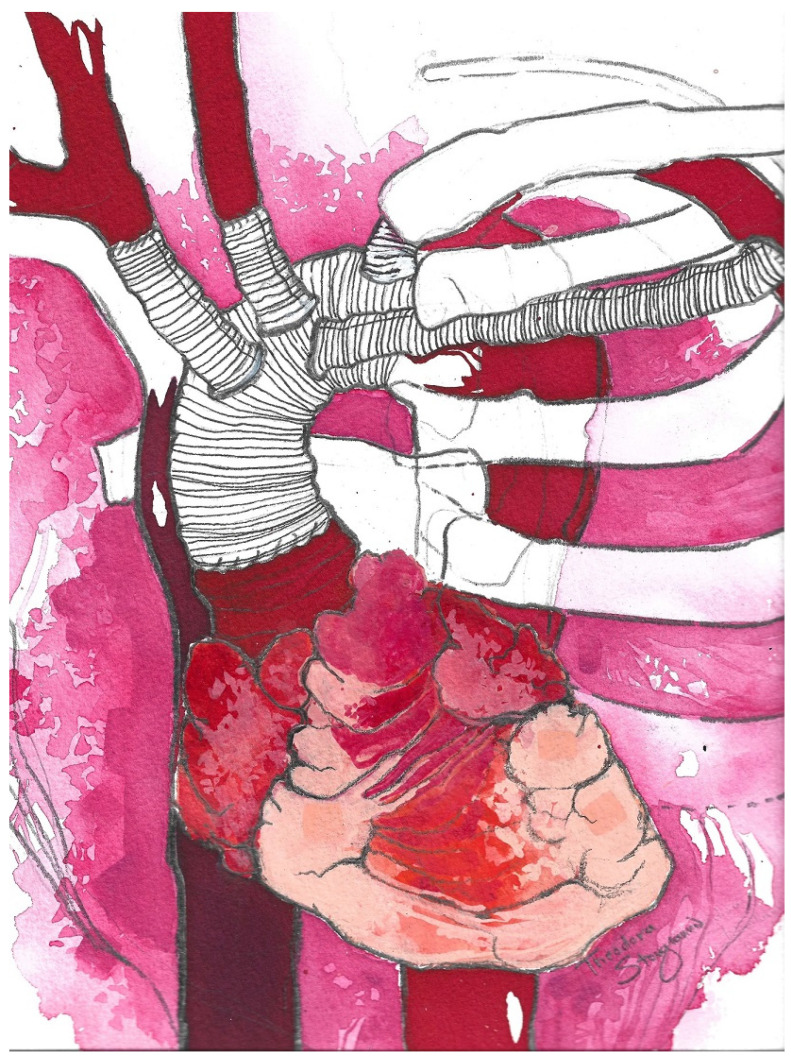
An illustration showing the extra-anatomic conduit created by anastomosing the perfusion side branch to the left axillary artery. The third branch of the Thoraflex^TM^ graft, along with the proximal end of the SC, are ligated (ligations situated behind the clavicle, not shown in the illustration).

Another valid technique which has been described before, and which we sometimes choose to perform as well, is the creation of an extra-anatomic circuit with the perfusion side branch of the Thoraflex^TM^ graft. The side branch is anastomosed to the left axillary artery over the second rib; in this scenario, the LSA is proximally ligated and the graft itself is passed through the left pleura and second intercostal space, beneath the pectoralis major muscle, ensuring the appropriate length and avoiding kinking or tension (Figure 5). Finally, spinal cord protection with a cerebrospinal fluid (CSF) drain should be employed in all elective cases, in the absence of any contraindications [26].

## 5. Sizing of the Graft

Although there is substantial available literature on the FET technique, it appears that no consensus yet exists on the correct hybrid graft sizing. The stented portion of the hybrid graft is designed to seal re-entry towards the descending thoracic aorta, in cases of AAD, by expanding the true lumen and thereby forcing the false lumen to eventually thrombose. However, this has been shown to cause further injury to the intima and promote endoleaks [27]. In general, most surgeons prefer not to oversize the stent–graft in AAD cases, with most groups utilizing the maximal true diameter for sizing [28,29]; still others use the aortic diameter before dissection as the criterion for the stent–graft sizing [30].

In chronic dissections, where the flap seems to be more rigid, many surgeons avoid oversizing, preferring to size the graft based on the maximum diameter of the true lumen, while in aneurysms, most surgeons agree on 10–20% oversizing, at about the level of the distal landing zone. Regarding the stent length, the risks related to endoleaks, associated with shorter stent lengths, must be counterbalanced with the risk of paraplegia brought on by longer stent lengths. Our approach is aligned with that of most centres on this matter, which dictates the use of a shorter device, accepting the risk of an endoleak and possible reintervention in the future [31]. As such, we opt to use a 100 mm graft and anastomose in zone 2, securing the stented part of the prosthesis above the level of the seventh thoracic vertebra (Th7); when a longer stent is required, we usually prefer to proceed with a second-stage endovascular procedure. Regardless of the graft’s size, a thorough assessment of the preoperative computed tomography angiogram is imperative [27].

## 6. Outcomes

Through use of the FET technique, aortic arch replacement procedures, especially when performed at high-volume centres, have been associated with very good outcomes. More specifically, Tian et al., in a very early meta-analysis, presented data on the technique’s safety and efficacy; their analysis, which included 17 observational studies, found a pooled mortality of 8.3%, a stroke rate of 4.9%, and a spinal cord injury rate of 5.1%. The five-year survival, as reported in only five studies, ranged between 63 and 88% [32]. Another study, from the UK aortic group, including eight high-volume centres in the UK deploying the Thoraflex^TM^ Hybrid graft in 66 patients suffering from AAD, reported an in-hospital mortality of 12% [33]. In this case, though no spinal cord injury events were documented, postoperative temporary or permanent neurologic events occurred in about 17% of the patients.

Similar results seem to have been presented in the paper of Jakob and colleagues, who collected data on 96 AAD patients treated with an E-vita^®^ Open hybrid graft [34]; in this study, the authors presented a 12% hospital mortality with a 7% risk of postoperative stroke and a 5% risk of spinal cord injury. Shrestha and colleagues reported similar data from their experience using the Thoraflex^TM^ Hybrid graft on 100 patients (37 acute dissections, 31 chronic dissections, and 32 aneurysms). According to this study, the rates of stroke, paraparesis, and recurrent laryngeal nerve palsy seemed to be at about 9%, 7%, and 25%, respectively, with the acute kidney injury risk at about 30%, and perioperative mortality at 7% [31]. The complication rates for chronic dissections and aneurysms were lower than those for AAD patients, as anticipated. The same group further presented their single-centre experience with 251 patients over 15 years with multiple prostheses, reporting a 30-day mortality rate of 11% for aneurysms and AADs, and 4.3% for chronic dissections, with stroke rates of 13.4%, 18%, and 10% for each of the three groups, respectively. During the long-term follow-up period, 26% of the patients with an aneurysm needed a secondary procedure in the descending aorta, though AAD patients seemed to exhibit stable aortic diameters within the stented segments, a finding that, according to the authors, confirms the post-FET remodelling of an acutely dissected aorta. As far as the chronic dissection patients were concerned, 26 out of 66 patients required reoperations in the downstream aorta [35]. Despite the fact that there is a dearth of literature regarding the long-term outcomes of the FET, the prospective analysis of Verkstein et al., covering a period of 17 years, exhibited promising results in terms of the overall survival (54% at 10 years) and need for reintervention (7%) [36].

In a more recent study, but with a small patient cohort of 50 patients undergoing FET for an AAD, aneurysm, or chronic dissection, Shimamura et al. reported a 30-day and in-hospital mortality of 2%, as well as stroke and neurologic deficits at around 2% and 6%, respectively; the survival rate at 1 year, 2 years, and 5 years seemed to be at 96%, 92%, and 85%, whilst freedom from an unplanned distal reintervention at 1 year, 2 years, and 5 years seemed to be at 98%, 92%, and 81%, respectively. In the same study, a CT follow-up over 5 years demonstrated positive distal aortic remodelling with aneurysmal regression and stable aortic dimensions in AAD patients, which seems to be in line with previous studies [35,37].

Leone et al., in their study of 437 patients who underwent a total arch replacement using the FET technique, presented an overall in-hospital mortality of 14.9%, while a permanent neurologic deficit and spinal cord injury seemed to be at 10.8% and 5.5%, respectively. Interestingly, in this cohort, the patients with a chronic aortic dissection did better, in terms of the in-hospital mortality rate, compared to the TAA and AAD groups. The authors attributed this to the poorer overall status and more severe atherosclerotic pathology commonly encountered in patients suffering from aneurysmal disease. In a midterm follow-up of 3 years, 23.1% required an additional procedure, 16.3% required endovascular extensions, and 6.7% required aortic surgery [38]. With regards to the descending aorta (DA), the French registry, using the E-Vita Open Plus, reported rates of 10.8% for endovascular completion, and 3.6% for secondary surgical aortic procedures, in elective surgery patients with a chronic dissection and aneurysm after only 1 year [39]. Finally, Ma and colleagues, in their analysis of the data on 518 AAD patients, showed favourable early and long-term survival, as well as freedom from reoperation for up to 15 years. The authors further reported an operative mortality of 7.5%, while late survival and freedom from distal reoperation were at 77.3% and 69.8%, respectively [40].

### 6.1. Aortic Remodelling

The term aortic remodelling was introduced in 2017 by Iafranesco et al. to portray the diameter and volume changes in the true and false lumen across the length of a dissection [41]. The Society of Vascular Surgeons and the Society of Thoracic Surgeons highlight the significance of the “10% rule” for defining and classifying aortic remodelling into three distinct strata: positive, stable, and negative. Positive remodelling refers to an increase in the aortic true lumen ration (TLR) > 10%, with a stable aortic lumen or a decrease > 10% of the lumen with a stable TLR. If these changes do not surpass 10%, then remodelling is deemed as stable, while a substantial increase in the aortic lumen or a decrease in the true lumen’s diameter are typical of a negative remodelling [42]. However, these definitions may sometimes lead to misconceptions, particularly when both positive and negative changes are observed across different parts of the aorta. To address this limitation, studies have supported the notion of measuring the diameter across different levels of the DTA [43]. A recent study from the Netherlands defined positive aortic remodelling as false lumen stability with at least partial thrombosis, the absence of overall diameter advancement, and no need for reintervention on the downstream aorta [44].

It seems that there is a controversy between studies regarding remodelling in acute and chronic dissections. A multicentre study including almost 400 patients treated with an FET reported no differences in the remodelling between the two clinical entities [41], while others have demonstrated a favourable remodelling in acute dissections [43]. During the first year after a FET procedure, these changes are typically restricted to the level of the stent graft and, thus, little is known about the aortic remodelling of more distal sections of the aorta. The follow-up data have shown that up to 40% of cases have reported negative remodelling mandating distal re-intervention to prevent rupture [45]. Approximately one-third of these patients will undergo a left thoracotomy or resternotomy due to a patent false lumen [46]. Subsequently, the long-term follow-up data have shown that a patent false lumen, especially in those with a preoperative descending thoracic aorta > 40 mm, was associated with higher rates of 10-year reoperation [47].

### 6.2. FET as a Redo Operation

To what extent the index aortic event is repaired potentially affects the risk for reintervention. The untreated segments of an aorta may lead to a newly occurring (rupture or dissection) or a progression of the existing aortic pathology. The evidence suggests that a limited repair has better short-term outcomes, but may necessitate a more complex secondary intervention. Hence, the scope of the initial repair remains controversial, primarily due to concerns about the high mortality rates of a redo aortic arch repair [48]. The aging of the general population, the improved survival of patients undergoing an “initial” aortic arch surgery, and the meticulous follow-up protocols may account for the increased number of aortic arch reoperations observed over the last years [49].

A hemiarch replacement is probably the most employed treatment choice in patients presenting with type A AAD. However, one-fourth (24%) of these patients will require a subsequent reintervention within 10 years because of the underlying disease’s progression. In these cases, the benefits of an endovascular repair must be weighed against those of an open surgical arch replacement [50]. A multicentre study showed that only 70% of patients qualify for an endovascular reintervention, primarily due to the shortness or kinking of the grafts, with the rest of the cases being treated/handled with an FET [51]. In general, the main indications for a redo FET procedure are as follows: an endoleak with a sac expansion; pseudoaneurysm or anastomotic leaks; postoperative infections with/without fistulae formation; and the need for concomitant procedures in the ascending aorta, cardiac valves, or coronary arteries. A redo surgery poses unique challenges, demanding advanced imaging techniques, and revolutionary and tailored surgical approaches by a multidisciplinary and highly trained team to ensure optimal outcomes [52].

A German analysis of 237 patients undergoing an elective redo FET to treat residual type A aortic dissections following a hemiarch aortic replacement showed encouraging results. Over a median postoperative period of 14 months, 40 (16.8%) patients died (15 during the in-hospital period), while 18 experienced a stoke [49]. Similarly, Demal et al. highlighted an elective FET as a viable and safe option for a redo procedure in relatively young patients with a residual dissection or progressive thoracic aneurysmal formations following a previous aortic surgery; most of the cases had initially undergone a supracoronary ascending/hemiarch replacement. In their analysis, an FET was employed either as the primary procedure or for a reoperation, yielding similar results for the mortality (7.4% vs. 3.2%, respectively) and postoperative complications in both groups, such as acute renal failure (18.5% vs. 16.1%) and neurological deficits (9.3% vs. 6.5%) [50].

Furthermore, Berger and colleagues described the outcomes of using the FET technique after a previous proximal or even distal (open or endovascular) thoracic aortic repair. The in-hospital mortality was 3% and the incidence of stroke was 8%, while 63% of the patients were free of any reintervention during a median follow-up of 18 months. The cohort was characterized by high rates of connective tissue disorders and genetic aortic syndromes. This finding may be explained due to the known risk of aortic diameter progression in untreated segments, often leading to multiple reinterventions following proximal repairs. When that is the case, an endovascular repair of the descending aorta is generally discouraged; however, the adoption of the FET technique has shown excellent outcomes and any required more distal extensions can be safely addressed by open surgery, thoracic endovascular aortic repair (TEVAR), or a combination of the above [48]. However, despite the increasing amount of data on the use of the FET in patients with a previous cardiovascular surgery, the optimal treatment modality is yet to be decided, since prospective studies making a direct comparison between endovascular aortic arch repair and an FET are lacking.

## 7. Discussion

The utilization of hybrid prostheses in the FET technique is one of the most prominent advancements in surgery on the aortic arch, considered a game changer by many. In general, all elective cases require multidisciplinary input and meticulous planning. Hybrid prostheses have a unique versatility, while the FET technique itself is reproducible as well. As with most sophisticated surgical techniques, the FET also requires careful patient selection, especially in the early stages; as a surgeon’s and centre’s experience grows, it is thought that eventually the indications may expand to include patients with more complex anatomy, as well as to patients presenting with acute aortic syndromes. Unequivocally, the FET technique will continue to gain in popularity, as it confers many benefits; it facilitates single-stage repair, promotes aortic remodelling in patients with AAD, and simplifies distal reintervention for TEVAR. In fact, the FET serves as a linkage between traditional open surgery and endovascular techniques, since its graft design creates an ideal landing zone for secondary TEVAR, allowing for the treatment of even extensive thoracoabdominal clinical entities [53,54]. A recent retrospective study showed the feasibility of TEVAR following an FET, exhibiting an absolute technical success rate (100%) and a 95% survival rate over a follow-up of approximately 60 months [55]. Such devices have shown promising outcomes with relatively few complications and low mortality rates, compared to conventional challenging aortic arch replacement procedures, which have frequently been associated with serious mortality and morbidity rates.

It is thought that more than 30,000 hybrid prostheses have been implanted worldwide thus far, with an early mortality ranging from 1.8% to 17.2% [56]. The data specifically for Thoraflex^TM^ are very encouraging, since Tan et al., in their analysis of 931 patients from various aortic centres, showed an overall mortality of 1.5%, with a 7-year survival rate of 99%, and with freedom from adverse events at 95% at 84 months. Despite the many favourable features of Thoraflex^TM^ and the FET in general, it may also be associated with disadvantages, including a risk of neurological complications; this comes as no surprise, as this method employs a period of circulatory arrest, long CPB times, and cerebral perfusion techniques, with graft sizing and positioning within the descending aorta, which may impede spinal cord perfusion. Consequently, stroke, TIA, cognitive decline, delirium, and paraplegia may develop [57]. More specifically, the rate of paraplegia in recent meta-analyses seems to be around 5 to 10% [58,59], though some groups have reported higher rates with previous generation grafts, including the E-Vita^®^ Open, which seem to be as high as 19.6% [60].

Another major complication, related to CPB and the hypothermic circulatory arrest often used in aortic surgery, is the development of coagulopathy, often leading to a reoperation for bleeding along with the transfusion of blood products. The association between the FET procedure and perioperative coagulopathy is well documented; Bashir et al., through a recent systematic review, attempted to interpret the observed coagulopathy complications after an FET, a study which included a total of 46 studies consisting of 6313 patients, collectively treated with four devices, namely the Thoraflex^TM^ Hybrid, E-vita^®^, Frozenix^®^ (Japan Lifeline Inc., Tokyo, Japan), and Cronus^®^ (MicroPort Medical (Shanghai) Co., Ltd., Shanghai, China). Through this study, the authors reported a rate of 17% for postoperative bleeding amongst the patients included, while the pooled estimate of reoperation was at about 7%. This meta-analysis further revealed that rate of reoperation for postoperative bleeding was the lowest for the Thoraflex^TM^ Hybrid and Frozenix^®^, while an additional subgroup analysis revealed no heterogeneity between the Thoraflex^TM^ Hybrid and Frozenix^®^, as opposed to the moderate heterogeneity observed during a comparison of the E-vita^®^ and the Cronus^®^ devices. In this regard, the Thoraflex^TM^ seems to have fared well against the competition, as shown in multiple, published studies [61].

Regarding the E-vita^®^ Open, Czerny et al., in their recent study, reported excessive oozing through the stent fabric in three patients who received the branched E-vita^®^ Open NEO hybrid prosthesis [62]. The E-vita^®^ Open NEO HP has also been shown to exhibit a propensity for excessive oozing through the stent fabric, specifically in the aortic arch portion composed of polyester material, as this area lacks gelatin, increasing the porosity, and thus rendering it more susceptible to bleeding [61]. The issue of excessive oozing with the E-vita^®^ Open NEO has also been raised in a report by Ho et al., though the safety of the attempted, pre-emptive use of BioGlue on the stent, as priming before surgery to prevent oozing and mitigate this effect, seems questionable and has not been proven with relevant studies [63].

On the other hand, Berger and colleagues, evaluating early and mid-term outcomes, as well as aortic remodelling in 88 AAD patients, have shown a significantly higher rate of secondary endovascular aortic interventions in the Thoraflex^TM^ group compared to the E-vita^®^ Open group (33 patients) (22% vs. 0%; *p* = 0.003), though the rate of spinal cord injuries remained the same. According to the authors, this may be attributed to the longer coverage of the descending aorta provided by the longer stent graft design, associated with the more frequent implantation within zone 3 for the E-vita^®^ Open group [64]. Another complication that is graft-related is a distal stent graft-induced new entry (dSINE). This is an established complication related to an FET and, in a recent systematic review, it was reported to have an overall incidence ranging between 0% and 27.3%. In this review, the Thoraflex^TM^ graft was reported to have an incidence of this complication from 0 to 14.5%, and the E-Vita of 1–18.2%. The dSINE is a rather serious and potentially detrimental complication, and factors, such as the type of the graft, the degree of oversizing, and the distal landing zone, have been suggested to increase it [65].

A recent meta-analysis of 28 studies including approximately 2200 patients treated with the E-vita or Thoraflex^TM^ (1919 vs. 242, respectively) reported an increased 30-day mortality, neurological deficit, and one-year mortality rates associated with the Thoraflex^TM^. However, the very small number of patients undergoing surgery with the Thoraflex^TM^, the higher proportion of non-elective operations in this cohort, and the highly heterogeneous studies did not allow for definitive conclusions to be drawn [54,66]. Another multicentre, though smaller, study of 88 patients undergoing an FET for AAD, reported at the 1-year follow-up a higher rate of endovascular extension with the Thoraflex^TM^ Hybrid prosthesis compared to the E-vita^®^ Open, with a rate of 22% for the former versus 0% for the later, respectively. This finding, along with a general trend of higher false lumen thrombosis rates for the E-vita^®^ Open, seems to be generally attributed to the longer stent graft design, hence the longer coverage in the descending aorta, as well as the more frequent implantation in zone 3 [64]. Subsequently, a retrospective study of patients undergoing an FET procedure reporting both early and late outcomes showed the superiority of the Thoraflex^TM^ in terms of the duration of CPB, myocardial ischemia, lower ventilation times, the on-set of kidney failure, paraplegia, and overall mortality compared to the E-Vita. On the other hand, freedom from TEVAR for up to 5 years following the initial procedure was lower in patients with the Thoraflex^TM^ [6].

## 8. Conclusions

The advent of the FET technique for complex aortic arch surgery has revolutionized the management of thoracic aortic disease. Cardiovascular surgeons have the potential to efficiently replace the aortic arch and stent the descending thoracic aorta in a single stage using sophisticated hybrid prostheses. The documented outcomes, even in this high-risk patient population, are favourable compared to traditional surgical techniques. In general, all the available evidence suggests that despite any observed problems, the FET technique is associated with acceptable mortality and complication rates. Although the pool of patients requiring an FET seems to be heterogenous, and there is also a well-documented diversity amongst FET devices, a well-powered, robust, randomized trial is required to further evaluate all these variables, so that the best device for each case may be determined.
